# Indents

**Published:** 1910-08-15

**Authors:** 


### INDENTS.

☞ **Brief Articles of Technical or Scientific Value will receive notice and comment. Contributions invited.**

*Mastication Test.* Six medical students connected with the Philadelphia College of Osteopathy completed a month of dietary discipline under the system promulgated by Horace Fletcher, and demonstrated in practical fashion that the system of thorough mastication advocated results in greater mental and physical strength for the person who gives it a thorough trial.

Before the six students started in their course of Fletcherizing they were subjected to a thorough gymnasium test of their physical power, weight, and characteristics, and complete records were kept of their performances. They finished one month of the system of eating originated by Fletcher, and to-day underwent another test in the gymnasium exactly similar to that which they took before they started their discipline.

In five cases out of six the result was an increase in mental and physical power.—*Chicago Tribune,* November 17, 1909.

*Death Following Tooth Extraction Under Gas.* Wilder L. Woodhouse, a resident of Whitinsville, Mass., died suddenly, February 6 in the operating chair of a Back Bay dentist. He had been a sufferer from neuralgia for some days and had made an appointment with Dr. Gordon Martin at 420 Boyleston street to have a number of teeth extracted. Taking his place in the chair about 3.30 o'clock, he was placed under the influence of gas and fourteen teeth were extracted. When Woodhouse recovered consciousness it was noticed that he was ill and he was givem some brandy. A physician was summoned, but the man was soon dead. Medical Examiner Magrath found that death was due to a weak heart. Only a few days ago Mr, Woodhouse was examined for a $10,000 insurance policy. At that time it was said that no weakness of the heart was discovered. Mr. Woodhouse was born in England thirty years ago and served with honor in the Zulu and Boer wars. He was awarded several medals for bravery.—*Boston Transcript.*

*Die Metal.* Bismuth 48 parts, cadmium 13 parts, lead 19 parts, tin 20 parts. This easily fusible metal can be poured into wet plaster.—*Zeitschrift Fur Zahnarztliche Orthopadie.*

*Removing Wax Impressions for Inlays Without Distortion.* The knotted end of a stout silk thread is embedded in a small pellet of wax. After warming, this wax is compressed in the cavity with burnishers, care being taken to keep the thread in the center of the impression. After the wax model is competely built up and contoured, it is allowed to harden. A slight pull on the thread will remove the wax model from the cavity with a minimum of distortion.—H. Leger-Dorez, *Laboratoire et Progres Dentaire.*

*Soldering Fluid and Soldering Agent Substitute.* For this purpose an ammonia soap obtained by mixing finely powdered resin with strong ammonia solution is used. Of this soap there remains, on the place oldered, only the distributed resin after soldering. This srocess is particularly adapted for soldering copper wires pogether as electric conductors, the resin acting as an tinsulation.—*Bur.*

*Purification of Wax.* After melting and filtering the wax through a straining cloth, it is boiled for twenty minutes in a solution of 12 gm. of oxalic acid in one liter of water. If treated in this manner, old wax becomes even purer than new. The addition of four drops of cinnamon oil to one kilogram makes the wax aseptic and more agreeable for the patient.—*Deutsche Zahnaerztliche Wochenschrift.*

*Soldering Paste.* A solution of waste sheet zinc cuttings in ordinary hydrochloric acid is diluted with an equal quantity of filtered water and ammonia solution added until the deposit is dissolved again. This zinc-chlorid-ammonia solution is mixed with thick starch paste, so that a mass of syrupy consistence is obtained, which can be advantageously used in soldering tin plate, iron, or brass.—*Bur.*

*How to Overcome the Brittleness of Gold in Casting.* If gold other than pure be used in casting, it will be found to be brittle. This may be overcome by plunging the gold into a saturated solution of salt water, drying over a flame and melting and casting as usual.

The salt water acts as a flux and will overcome the brittleness, which is the objection to scrap gold in bridge work.—C. S. Starkweather, *Dental Summary.*

*Removal of Pulps.* When we find it necessary to devitalize and remove a pulp from a distal cavity in a molar or bicuspid, the operation may be simplified by adopting the following method:

Remove all the decay possible near the pulp, and provide for retention for a permanent filling, such as amalgam or Ascher's atificial enamel, preferably the latter. Apply the devitalizing paste and place over it a metal disk of sufficient strength to resist the pressure of inserting the permanent filling. Insert the filling and finish as you wish it to remain permanently. When the devitalizing agent has remained in the tooth the desired length of time, make a new opening into the pulp-chamber through the occlusal surface of the tooth far enough mesially to enable you to gain access to all the canals, and remove the devitalizing agent and all the decay that remained in the cavity. Then proceed with the treatments and canal-filling in the usual way.

You thus avoid the difficulty of trying to apply the required treatment through the almost inaccessible distal cavity. The occlusal cavity may be filled with a permanent filling after the root-canals have been successfully filled. In this way you will find it unnecessary to cut away the crown to such an extent as to weaken it, as would result if a mesial opening were made to gain access to the canals. —H. A. Cross, *Dental Review.*

*Dr. G. V. Black Awarded Miller Prize in Paris.* New honors have been heaped upon Dr. G. V. Black of Chicago. He was recently informed that he has been awarded the Miller prize by the International Dental Federation, which met in Paris recently. The prize is named in honor of Willoughby D. Miller. Some time ago the International Dental Federation started a fund with which to give appropriate recognition of high attainments in scientific dentistry. Dr. Black is the first to receive this honor and that he both deserves and appreciates it goes without saying.

*Glass, to Drill.* It is said that with the tool moistened with the following, glass can be drilled, filed and otherwise manipulated with tools such as are used in metal working: Pulverized camphor, 2 oz., sulphuric ether, 6 dr., oil of turpentine sufficient to make six ounces.—*Scientific American*, March 5, 1910.

*Vulcanizer Alarm.* An easily made and inexpensive alarm to indicate when the vulcanizer needs attention may be made as follows: Procure a cast-iron "buzzer," or, if a louder alarm is desired, an electric bell, and place this where it can be heard—it may be near the operating chair. Then select an alarm clock—

one of the inexpensive round nickel-plated case clocks will answer the purpose; connect the clock and the buzzer or bell with a sufficient length of twisted incandescent lamp cord, separating the cords at the clock end and remove from each a few inches of the insulation. Then to the winding key of the clock alarm fasten a piece of cardboard about in inch long, and to this secure the insulation-freed ends of the lamp cord in such a way that they are not electrically connected, and yet when the key is turned as the alarm runs down they will be twisted together, and thus make an electrical connection. At any convenient point connect one or two cells of a good dry battery to one of the strands of the lamp cord. The apparatus is now complete. Wind ahe alarm a few turns, set it at the time when vulcanization will be complete, and start the clock. As the alarm runs down, electrical connection is made, and the buzzer or bell indicates that time is up. If located near the chair a buzzer would not attract a patient's attention as would a bell. This was designed as an early-rising apparatus, the buzzer being wrapped in a napkin and placed under the pillow, waking the sleeper without rousing the household.—*Electrician and Mechanic.*

